# Case Report: Transcatheter closure of paravalvular leak following left ventricular assist device and aortic valve replacement

**DOI:** 10.3389/fcvm.2026.1742624

**Published:** 2026-04-13

**Authors:** Ping-an Lian, Shuai Cheng, Shan-fu Liang, Fei Xie, Zhan-zhan Zhu, Yun-fei Zhao, Lei Yin, Shen-wei Zhang

**Affiliations:** 1 Department of Cardiology, Seventh People’s Hospital of Zhengzhou, Zhengzhou, Henan, China; 2 Institute of Biological Therapy, Henan Academy of Innovations in Medical Science, Zhengzhou, Henan, China; 3 Seventh People’s Hospital of Zhengzhou, Zhengzhou, Henan, China

**Keywords:** aortic valve replacement, heart failure, left ventricular assist device, paravalvular leak closure, transcatheter intervention

## Abstract

**Background:**

End-stage dilated cardiomyopathy (DCM) is a common cause of end-stage heart failure, and some patients may eventually require heart transplantation. Such patients often need a left ventricular assist device (LVAD) as a bridge to heart transplantation, and those with severe aortic regurgitation may undergo concurrent aortic valve replacement. Paravalvular leak (PVL) is a complication following valve surgery that can lead to worsening heart failure; however, reports on transcatheter closure of PVL after LVAD implantation are rare.

**Case summary:**

This article reports the case of a 39-year-old male patient with DCM who underwent CorHeart 6 LVAD implantation, aortic bioprosthetic valve replacement, and tricuspid valvuloplasty due to end-stage heart failure. A follow-up echocardiogram 2 months post-surgery revealed a progressively worsening paravalvular leak around the aortic bioprosthetic valve (maximum regurgitant jet width 3.4 mm, regurgitant jet area 20.5 cm^2^), accompanied by worsening symptoms of heart failure. Due to the extremely high risk of redo surgery (STS score 16.2%), a multidisciplinary team consisting of cardiac surgeons, interventional cardiologists, intensive care physicians, and echocardiographers decided to perform transcatheter closure. The procedure was successfully performed via a retrograde approach through the right femoral artery, guided by fluoroscopy combined with transthoracic echocardiography. A 10 × 8 mm ventricular septal defect occluder was used to successfully seal the leak. Intraoperative angiography and echocardiography confirmed the satisfactory position of the occluder with no residual shunt. At the 2-month and 6-month follow-up visits post-procedure, the patient's cardiac function had improved to NYHA class II, echocardiography showed no residual PVL, and the left ventricular dimensions were slightly reduced compared to previous measurements.

**Conclusion:**

This case suggests that transcatheter closure may be a safe and effective treatment option for high-risk patients who develop PVL following LVAD implantation combined with aortic valve replacement, providing a reference for the individualized management of similar complex cases.

## 1 Introduction

Dilated cardiomyopathy (DCM) is one of the common causes of end-stage heart failure, and some patients may eventually require heart transplantation (1). For patients with end-stage heart failure refractory to medical therapy, left ventricular assist devices (LVAD) offer an effective alternative to heart transplantation, especially given the limited availability of donor hearts (2). For DCM patients with severe aortic regurgitation, LVAD implantation is often performed in conjunction with aortic valve replacement surgery (3). However, paravalvular leak (PVL), as one of the common complications after valve surgery, is closely associated with the deterioration of heart failure, hemolytic anemia, and infective endocarditis (4, 5).

The traditional treatment for symptomatic PVL is primarily surgical valve repair or re-replacement (5). However, for patients with an implanted LVAD, the risk of reoperative sternotomy is significantly increased. For patients with high surgical risk and suitable anatomy, such as those with refractory hemolysis or heart failure (NYHA class III/IV), transcatheter PVL closure is a reasonable treatment option (6, 7). We report a case of an end-stage DCM patient who developed PVL after LVAD implantation and aortic valve replacement and was successfully treated with transcatheter closure, aiming to provide a reference for the treatment of similar patients.

## 2 Case description

### 2.1 Preoperative clinical data

**Figure 1:**
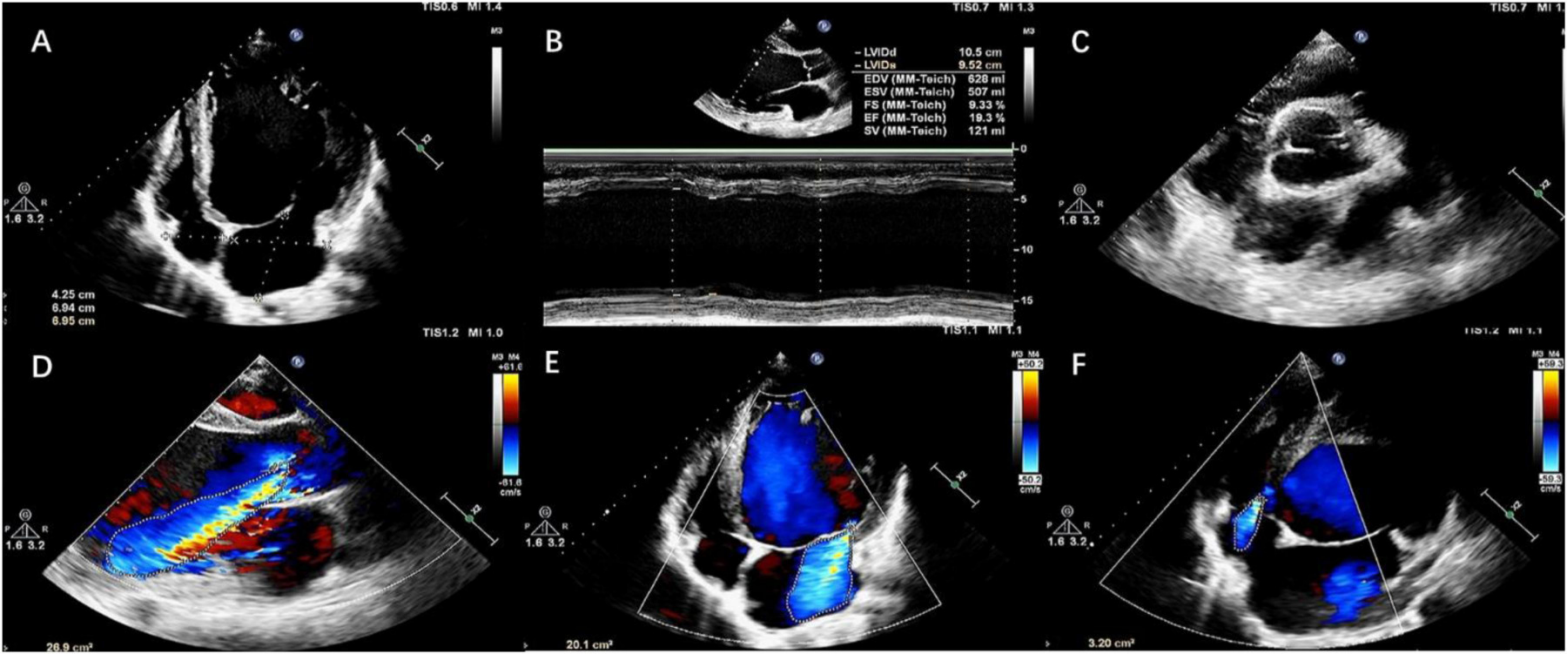
Preoperative transthoracic echocardiography findings. **(A,B)** Dilated left ventricle (LVEDd 105 mm), left atrium and reduced LVEF (19.3%). **(C)** The left and right coronary cusps of the aortic valve are fused, leading to functional bicuspid morphology. **(D–F)** severe eccentric aortic regurgitation, severe mitral regurgitation and mild tricuspid regurgitation.

A 39-year-old male patient was admitted to our center due to “dyspnea accompanied by lower limb edema for 10 years”. He had been diagnosed with DCM at an outside institution 10 years ago. Despite receiving guideline-directed medical therapy (GDMT), his condition continued to deteriorate and progressed to end-stage heart failure. At admission, the patient remained highly symptomatic, with persistent dyspnea (NYHA IV and INTERMACS 3). The serum NT-proBNP level was 20,147 pg/mL. Transthoracic echocardiography (TTE) revealed severe left ventricular (LV) dilation, with a left ventricular end-diastolic diameter (LVEDD) of 105 mm. The LV exhibited diffuse hypokinesis, and the left ventricular ejection fraction (LVEF) was 19.3% (modified Simpson's method). Right ventricular (RV) function was relatively preserved (TAPSE: 17 mm and FAC: 36%). The aortic valve showed fusion of the left and right coronary cusps, forming a functionally bicuspid valve. During diastole, malcoaptation of the valve leaflets led to a prominent eccentric regurgitant jet, measuring 26.9 cm^2^ in area, which was observed striking the anterior mitral leaflet. There was also severe mitral regurgitation and mild tricuspid regurgitation (Figure 1). The ECG showed sinus rhythm, with a QRS duration of 149 ms and no left bundle branch block (LBBB). Coronary angiography revealed no significant stenotic lesions. Cardiopulmonary exercise testing (CPET) showed a peak oxygen consumption (peak VO₂) of 15.3 mL/min/kg, which is 38% of the predicted value, meeting the indication criteria for heart transplantation.

### 2.2 Surgical procedure and early postoperative course

Conventional valve replacement and cardiac resynchronization therapy (CRT) were deemed to offer limited benefit. Given the scarcity of donor organs, and following a multidisciplinary evaluation by the departments of cardiothoracic surgery and cardiology, the patient underwent LVAD (CorHeart 6) (Table 1) implantation along with aortic valve replacement (Balance Medical A-25 bioprosthetic valve, 25 mm) as a bridging therapy to heart transplantation. Although TTE revealed severe mitral regurgitation, there was no evidence of valvular calcification, adhesion, or prolapse, suggesting functional regurgitation secondary to left ventricular dilation and mitral annular enlargement. Therefore, no surgical intervention was performed on the mitral valve. The right atrium was incised, and a saline test revealed tricuspid regurgitation, characterized primarily by annular dilation and malcoaptation of the anterior and posterior leaflets. To preserve right ventricular function, concomitant tricuspid annuloplasty (Edwards Lifesciences 4,900T annuloplasty ring, 34 mm) was performed. The LVAD pump speed was set to 2,700 RPM, with a flow rate of 3.9 L/min. Intraoperative transesophageal echocardiography (TEE) performed immediately after the procedure confirmed good function of the aortic bioprosthetic valve with no paravalvular leakage; only trace to mild regurgitation signals were observed in the mitral and tricuspid valves. Endomyocardial biopsy revealed diffuse myocardial fibrosis with mild lymphocytic infiltration, consistent with the pathological features of DCM. The symptoms improved significantly, with cardiac function improving to NYHA II-III.

**Table 1:** Summary of devices used in surgical and interventional procedures.

Device Type	Name/Specification	Manufacturer
Left Ventricular Assist System	Corheart 6	Core Medical
Aortic Bioprosthetic Valve	Artificial Biological Heart Valve (A-25)	Beijing Balance Medical
Tricuspid Annuloplasty Ring	Cardiac Annuloplasty Ring 4,900T (34 mm)	Edwards
Arterial Sheath	6F/7F Sheath	APT/Cordis
Angiography Catheter	5F Pigtail/6F AL1	Cordis
Guidewire (for crossing)	0.035-inch Hydrophilic Coated Guidewire (260 cm)	APT Medical
Guidewire (for support)	0.035-inch Amplatz Super Stiff (260 cm)	Boston Scientific
Delivery Sheath	7F Delivery System	Asia Pacific Medical
Occluder	VSD Occluder (10 × 8 mm)	Asia Pacific Medical

### 2.3 Diagnosis and progression of paravalvular leak

**Figure 2:**
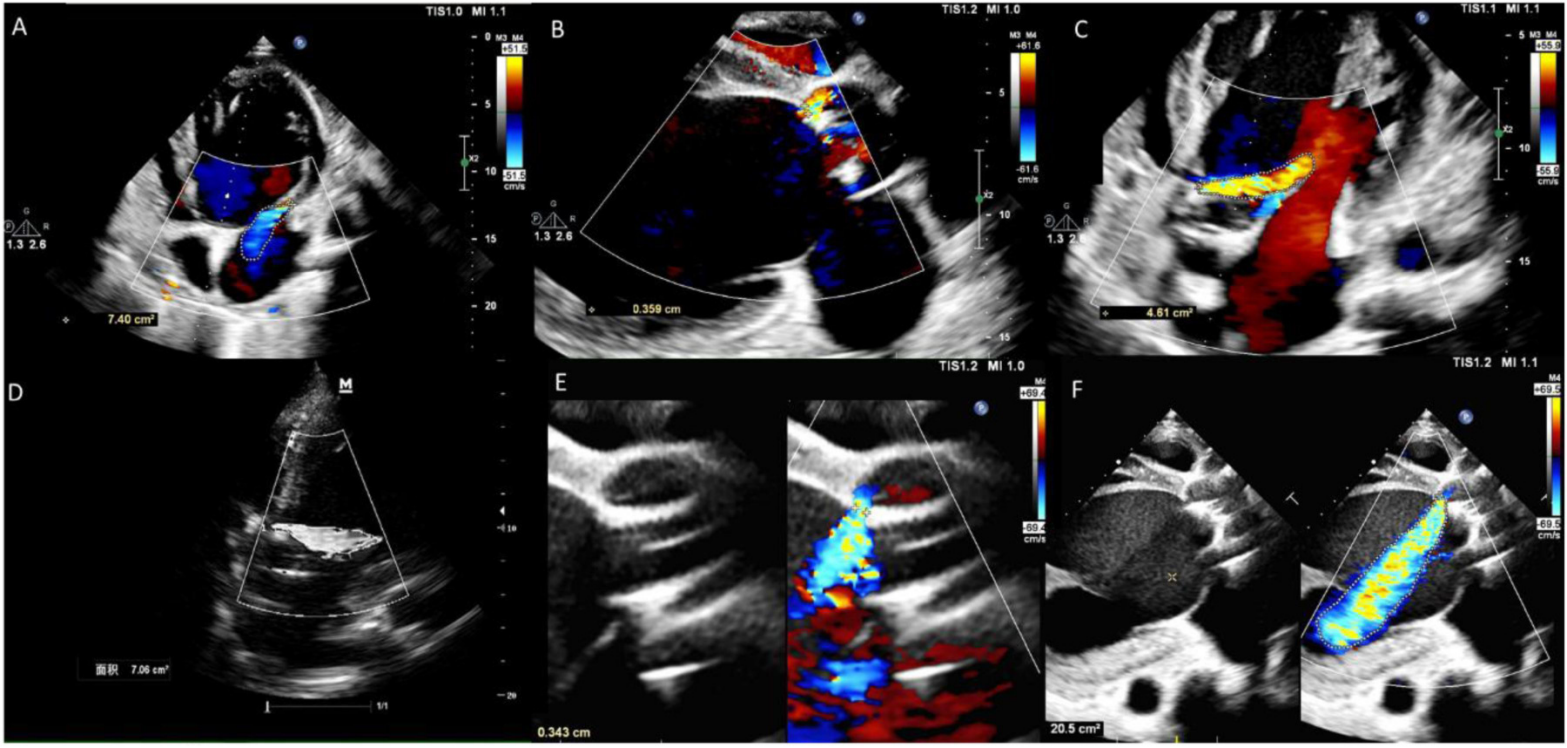
Development of paravalvular aortic regurgitation during follow-up. **(A–C)** Day 12 post-LVAD implantation, mitral regurgitation was markedly reduced; 3.6 mm paravalvular regurgitant jet was observed around the bioprosthetic aortic valve, with a regurgitant area of 4.6 cm². **(D)** Day 30 post-LVAD implantation, the aortic regurgitant area had increased to 7.1 cm². **(E,F)** At two months post-LVAD implantation, the paravalvular regurgitant jet measured 3.4 mm, with a significantly enlarged regurgitant area of 20.5 cm².

**Figure 3:**
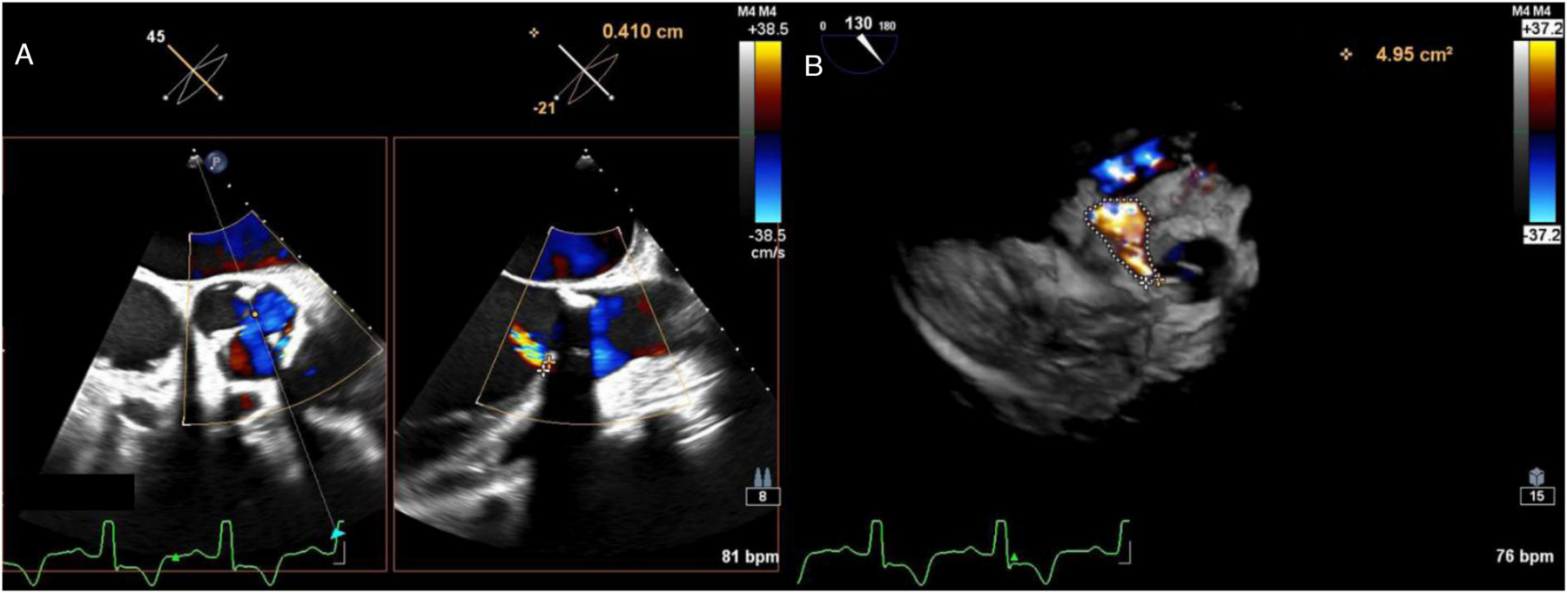
Transesophageal echocardiography identified the leakage orifice at the original right coronary cusp position, measuring 4.1 mm in width **(A)**, with a regurgitant jet area of 4.95 mm² **(B)**.

Postoperative TTE showed a significant reduction in mitral regurgitation, but a paravalvular leak was suspected around the aortic bioprosthetic valve. During the 2-month follow-up, repeat TTE revealed progressive worsening of the paravalvular regurgitation, with a maximum jet width of 3.4 mm and a maximum regurgitant area of 20.5 cm² (Figure 2). TEE further confirmed that the leakage was located at the original right coronary sinus of the aortic bioprosthetic valve, presenting as a localized single leak with a width of 4 mm (short axis) and a regurgitant jet area of 4.9 mm² (Figure 3). Despite stable LVAD pump flow, the effective forward blood flow and organ perfusion decreased, and the patient experienced worsening exertional dyspnea. Multiple objective evidence suggested that this PVL had a significant hemodynamic impact: (1) LVAD parameter changes: With the progression of the PVL, to compensate for the decrease in net cardiac output caused by regurgitation, the LVAD pump speed needed to be increased from a baseline of 2,700 RPM to 3,000 RPM before closure, with a corresponding increase in power consumption from 3.2 W to 4.0 W, while the stable flow showed a decreasing trend (from 3.9 L/min to 3.5 L/min) (Table 2). (2) NT-proBNP trend: Within the 2 months before closure, NT-proBNP showed a progressive increasing trend (from 4,230 pg/mL to 7,568 pg/mL), indicating worsening volume overload. (3) Left ventricular dimensions: Although more than 2 months had passed since LVAD implantation, the left ventricle remained significantly dilated, with no obvious reverse remodeling observed.

**Table 2:** Perioperative LVAD parameter changes.

Time Point	Pump Speed (RPM)	Flow (L/min)	Power (W)
LVAD Post-implantation Baseline	2,700	3.9	3.2
Pre-PVL Closure (at symptom worsening)	3,000	3.5	4.0
During PVL Closure	3,000	3.5	4.0
Post-PVL Closure (before discharge)	2,897	3.9	3.4

Due to the extremely high risk of redo surgery (STS predicted risk of mortality, 16.2%), a multidisciplinary team discussion (including cardiac surgery, interventional cardiology, intensive care, and echocardiography) led to the decision to perform transcatheter PVL closure.

### 2.4 Procedural details of transcatheter closure

**Figure 4:**
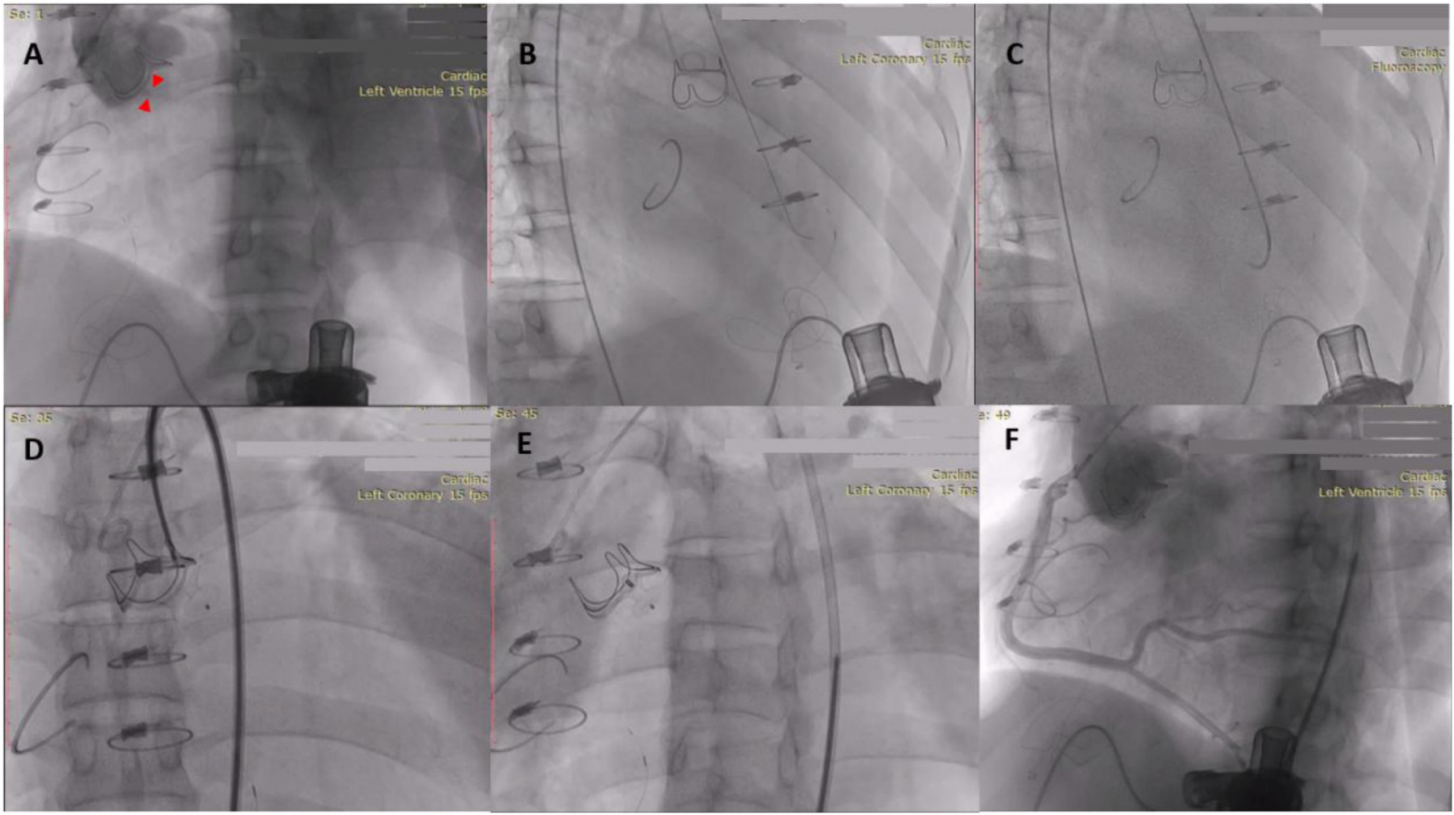
**(A)** Aortic angiography revealed paravalvular regurgitation; **(B)** guidewire crossing the PVL; **(C)** Exchange for the stiff guidewire via the 6F AL1 angiographic catheter; **(D,E)** the occluder was well positioned; **(F)** Angiography showed no residual paravalvular aortic regurgitation, and the occluder did not affect the valve opening or the right coronary ostium.

The procedure was performed under local anesthesia, guided by fluoroscopy combined with TTE. Preoperative CT was not performed because TEE had already clearly identified the location (original right coronary sinus) and size (4 mm) of the leak, which was sufficient to guide the selection of the occluder. The intraoperative procedural steps were as follows:

Arterial access establishment: A 6F sheath was inserted into the right radial artery as the access route for aortography, and a 7F sheath was inserted into the right femoral artery as the main procedural access route. Aortography: A 5F Pigtail catheter was advanced through the 6F radial sheath to the aortic root for angiography to preliminarily confirm the location and severity of the PVL (Figure 4A).

PVL Crossing: First, under fluoroscopy via the femoral artery approach, a 0.035-inch hydrophilic guidewire (APT Medical, 260 cm) was used to probe the leak. Preliminary localization of the guidewire trajectory was performed mainly using the right anterior oblique (RAO) projection combined with the caudal projection. When the guidewire was suspected of entering the left ventricle under fluoroscopy (Figure 4B), TTE left ventricular short-axis view was immediately used to confirm whether the guidewire tip was located outside the annulus (i.e., perivalvular). After confirming the guidewire had correctly crossed the PVL, a 6F AL1 angiographic catheter (Cordis) was advanced over the guidewire through the leak into the left ventricular outflow tract.

Guidewire Exchange and Support Establishment: Through the 6F AL1 catheter, the hydrophilic guidewire was exchanged for a 0.035-inch Amplatz Super Stiff guidewire (Boston Scientific, 260 cm, standard J-tip). The guidewire tip was strictly controlled within the left ventricular outflow tract (LVOT) to avoid entering the apex and potentially interfering with the LVAD inflow cannula (Figure 4C). Resistance during advancement of the delivery system was minimal, and the guidewire remained stable; therefore, an arterio-arterial rail was not established.

**Figure 5:**
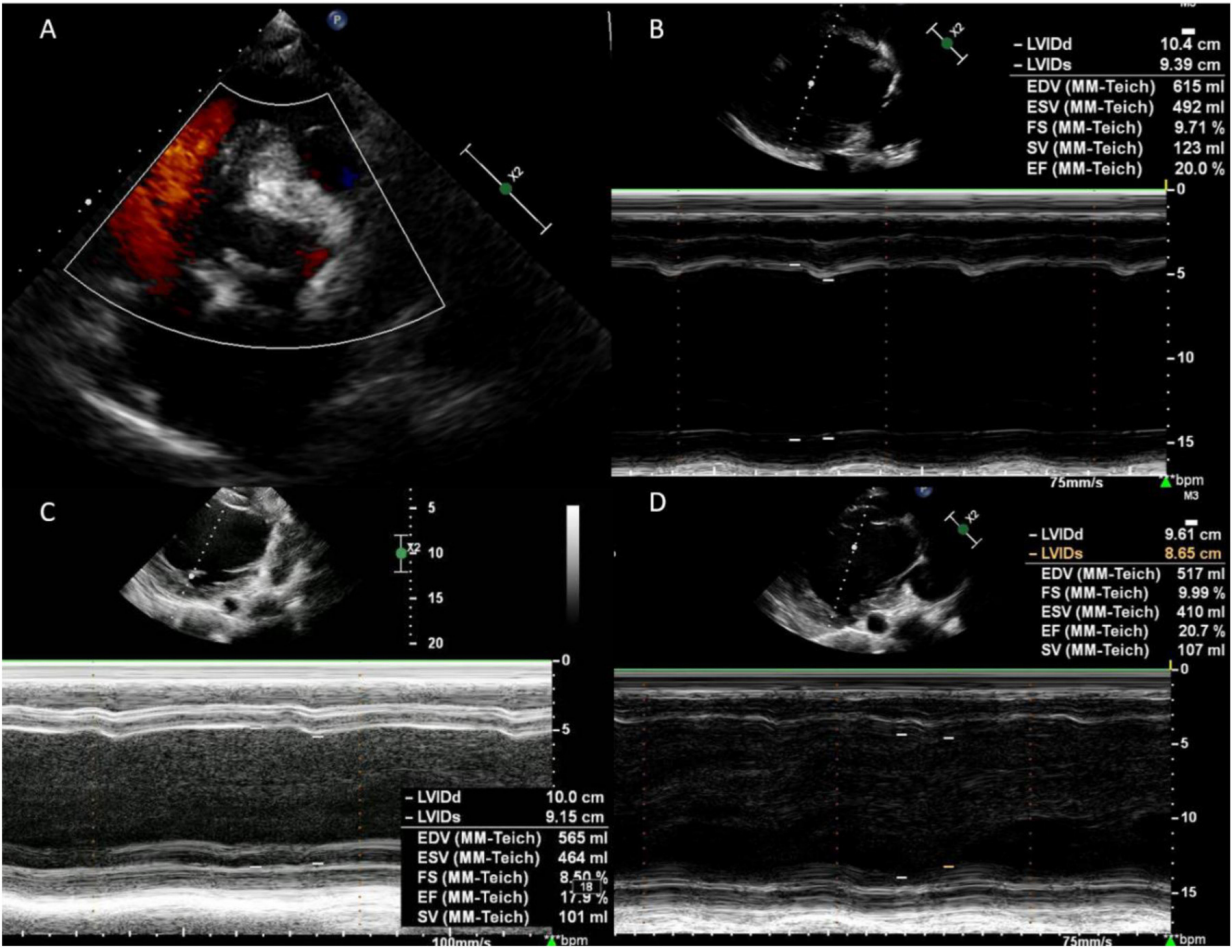
**(A)** Postoperative aortic short-axis view showing the occluder in a satisfactory position with no residual shunt on color Doppler. **(B)** Postoperative LVEDD 104 mm; **(C)** LVEDD 100 mm at 2 months postoperatively; **(D)** LVEDD 96 mm at 6 months postoperatively.

Occluder Delivery and Deployment: A 7F delivery sheath was advanced over the stiff guidewire. A 10 × 8 mm ventricular septal defect occluder (Asia Pacific Medical) was selected and deployed under fluoroscopic and TTE guidance. The left ventricular disc was deployed first, followed by retraction of the sheath to deploy the aortic disc. Final aortography and TTE confirmed satisfactory occluder position with no residual shunt and no impingement on aortic valve leaflet motion or coronary artery ostia (Figures 4D–F, Figure 5A). LVAD parameters were continuously monitored during the procedure; flow remained stable at 3.5 L/min with a power of 4.0 W, showing no significant fluctuations, thus no adjustment to the LVAD speed was made.

### 2.5 Postoperative follow-up

Postoperatively, the patient continued to receive standard heart failure medication therapy and warfarin anticoagulation (target INR 2–3). At the 2-month postoperative follow-up, TTE showed no residual paravalvular regurgitation, a slight decrease in left ventricular dimensions (LVEDD 104 mm → 100 mm) (Figures 5B,C), and NT-proBNP had decreased to 2,357 pg/mL. The patient experienced significant improvement in dyspnea upon exertion, with cardiac function improving to NYHA class II. At the 6-month postoperative follow-up, TTE showed a stable occluder position with no residual shunt, further reduction in left ventricular dimensions (LVEDD 96 mm) (Figure 5D**)**, and cardiac function remained at NYHA class II.

## 3 Discussion

This case report presents a patient with end-stage DCM, who developed PVL after undergoing LVAD implantation and bioprosthetic aortic valve replacement. The leakage was successfully treated via transcatheter closure. After heart valve surgery, approximately 2%–18% of patients may develop significant PVL, with the mitral valve and mechanical valves being more commonly affected (7, 8). Several surgical factors, such as the type of valve, implantation position, and suturing technique, contribute to an increased risk of PVL: mechanical valves present a higher risk than bioprosthetic valves; supraannular implantation carries a higher risk than intraannular implantation; and continuous suturing increases the risk compared to interrupted suturing (5). Other risk factors include annular calcification, tissue fragility, a history of endocarditis, or steroid use (9).

### 3.1 Rationale for PVL closure: objective hemodynamic evidence

Although the incidence of PVL is lower with bioprosthetic valves than with mechanical valves, it can still occur due to factors such as valve anatomy, left ventricular remodeling, and annular calcification. In this case, the patient developed a PVL following LVAD implantation combined with aortic valve replacement, which progressively worsened over a short period, leading to worsening heart failure. We emphasize that the indication for PVL closure should not be based solely on the size of the leak but rather on a comprehensive multi-parameter assessment. In this case, multiple objective pieces of evidence supported the rationale for closure intervention. First, LVAD parameters provided direct evidence of circulatory efficiency. Before closure, maintaining cardiac output required increasing the pump speed from 2,700 RPM to 3,000 RPM; however, the calculated flow actually decreased (3.9 → 3.5 L/min), and power consumption remained persistently high (4.0 W). This “pump speed-flow paradox” strongly suggested that part of the blood pump's output was being “wasted” by the PVL regurgitation. After closure, with a lower pump speed (2,897 RPM) and lower power consumption (3.4 W), the flow recovered to 3.9 L/min, indicating significantly improved circulatory efficiency.

Second, markers of neurohormonal activation and ventricular remodeling supported the diagnosis of worsening volume overload. NT-proBNP showed a progressive increasing trend before closure, reflecting continuously increasing right heart and volume load. The significant decrease in NT-proBNP after closure confirmed the hemodynamic improvement. Concurrently, left ventricular dimensions showed a consistent decreasing trend after closure (104 mm → 100 mm → 96 mm), consistent with the expected unloading of the ventricle. Third, the improvement in clinical symptoms aligned with the objective findings. The patient experienced progressively worsening dyspnea on exertion before closure (NYHA class III-IV), which improved to class II by 2 months after closure, further corroborating the clinical benefit of the intervention.

### 3.2 Potential mechanisms of PVL progression in LVAD patients

In this case, intraoperative TEE did not reveal an obvious PVL, yet significant PVL progression was observed at the 2-month follow-up post-surgery. This phenomenon suggests that the development and progression of PVL may result from multiple contributing factors, among which hemodynamic changes in the aortic root induced by continuous LVAD support may have played a crucial role.

First, the continuous, non-pulsatile flow pattern of the LVAD alters the pressure environment in the aortic root. In the absence of or with significantly reduced native cardiac ejection, the LVAD maintains forward flow during both systole and diastole, leading to a marked increase in diastolic pressure within the aorta. This elevates the pressure gradient across and around the valve on one hand, while on the other hand, it imposes continuous, cyclic mechanical stress on the bioprosthetic sewing ring and periprosthetic tissues. This stress may cause initially minute, intraoperatively undetectable potential gaps (possibly originating from suture needle holes, minor annular calcifications, or areas of tissue fragility) to gradually enlarge, forming a manifest PVL.

Second, during the follow-up period in this case, the aortic valve remained open (with TTE confirming good leaflet mobility), meaning that both LVAD flow and native cardiac ejection acted upon the aortic root, further complicating the hemodynamic environment. With LVAD support, the aortic valve may remain continuously closed or partially open throughout the cardiac cycle, potentially allowing PVL regurgitation to occur throughout the entire cycle (rather than being limited to diastole), thereby exacerbating the regurgitant volume load.

Third, echocardiography may underestimate the severity of regurgitation in LVAD patients. In the absence of effective native cardiac contraction, the pressure gradient between the left ventricle and the aorta is primarily generated by the LVAD. The regurgitant jet may be continuous and low-velocity, resulting in a diminished turbulent signal on color Doppler and thus underestimating the actual regurgitant volume. Although detailed hemodynamic simulation was not performed in this case, the persistently rising NT-proBNP levels and the lack of significant ventricular size reduction indirectly suggest that the actual regurgitant load may have exceeded the severity observed on echocardiography.

In summary, we hypothesize the following mechanism for PVL progression in this case: Initially minute gaps (possibly related to suture technique) gradually enlarged under the impact of continuous, high-stress flow from the LVAD; the elevated aortic diastolic pressure caused by the LVAD and possible pan-cyclic regurgitation aggravated the volume load; and conventional echocardiography may have underestimated the actual severity of regurgitation, leading to delayed clinical recognition. This hypothesis underscores that even “mild” PVL in LVAD patients warrants vigilance regarding its potential for progression, and its hemodynamic impact should be evaluated using a multi-parameter approach.

### 3.3 Technical considerations and lessons learned

Traditional surgical interventions pose extremely high risks for LVAD patients. Observational studies have shown that, compared to surgery, transcatheter closure for symptomatic PVL is associated with lower early mortality, while long-term mortality rates remain comparable (4). Therefore, when symptoms or hemodynamic instability caused by PVL, guidelines suggest that transcatheter PVL closure should be considered (10, 11). To the best of our knowledge, reports on the successful transcatheter closure of PVL occurring after LVAD implantation combined with aortic bioprosthetic valve replacement remain limited in the literature. This case provides a reference for the individualized management of similar complex situations.

In this case, the closure was performed via a retrograde right femoral artery approach, guided by fluoroscopy combined with TTE. Compared to conventional TEE guidance, TTE guidance avoids the need for general anesthesia and simplifies the procedural workflow, but it requires the operator to be proficient in the correlation between echocardiographic views and fluoroscopic projections. The experience summarized by our center is as follows: First, the guidewire trajectory is explored under fluoroscopy; then, the TTE short-axis view is used to confirm the guidewire is located outside the annulus (i.e., perivalvular). The correlation between these two modalities allows for accurate localization of the PVL. Regarding guidewire manipulation, strictly controlling the tip of the supportive guidewire within the left ventricular outflow tract, avoiding entry into the apex, is crucial to prevent interference with the LVAD inflow cannula. In this case, the Amplatz Super Stiff guidewire provided sufficient support, making the establishment of an arterio-arterial rail unnecessary; however, this option should be considered as a backup plan in the preoperative strategy.

### 3.4 Limitations

This case has the following limitations: First, as a single case report, the experience is difficult to generalize widely, and long-term follow-up data are lacking. Second, due to device limitations, the CorHeart 6 LVAD does not provide pulsatility index data, which restricted more detailed hemodynamic analysis. Third, invasive hemodynamic monitoring and serial lactate measurements were not performed. Fourth, preoperative CT assessment was not conducted, as TEE provided sufficient information to guide closure; however, CT is indeed a more standard method for PVL planning. Fifth, the echocardiographic assessment of regurgitation severity primarily relied on the regurgitant jet area rather than more recommended parameters such as EROA and PHT, which is an inherent limitation of this retrospective study. Future research needs to further explore optimal assessment strategies and technical specifications for PVL closure in LVAD patients.

## 4 Conclusion

This case report describes a patient who developed a PVL following LVAD implantation combined with aortic bioprosthetic valve replacement and was successfully treated with transcatheter closure. This case suggests that transcatheter closure may be a feasible treatment option for patients with PVL after LVAD surgery who are at extremely high risk for redo surgery, providing a clinical reference for the individualized management of such complex patients.

## Data Availability

The original contributions presented in the study are included in the article/Supplementary Material, further inquiries can be directed to the corresponding authors.
